# Early Stages of Alzheimer's Disease: Evolving the Care Team for Optimal Patient Management

**DOI:** 10.3389/fneur.2020.592302

**Published:** 2021-01-22

**Authors:** James E. Galvin, Paul Aisen, Jessica B. Langbaum, Eric Rodriguez, Marwan Sabbagh, Richard Stefanacci, Robert A. Stern, Elizabeth A. Vassey, Arno de Wilde, Neva West, Ivana Rubino

**Affiliations:** ^1^Comprehensive Center for Brain Health, University of Miami Miller School of Medicine, Miami, FL, United States; ^2^USC Alzheimer's Research Institute, San Diego, CA, United States; ^3^Banner Alzheimer's Institute, Phoenix, AZ, United States; ^4^University of Pittsburgh School of Medicine, Pittsburgh, PA, United States; ^5^Cleveland Clinic Lou Ruvo Center for Brain Health, Las Vegas, NV, United States; ^6^Thomas Jefferson University, Philadelphia, PA, United States; ^7^Boston University Alzheimer's Disease Center, Boston University School of Medicine, Boston, MA, United States; ^8^Alzheimer Center Amsterdam, Department of Neurology, Amsterdam Neuroscience, Vrije Universiteit Amsterdam, Amsterdam UMC, Amsterdam, Netherlands; ^9^Biogen, Cambridge, MA, United States

**Keywords:** Alzheimer's disease, dementia, early detection, diagnosis, mild cognitive impairment

## Abstract

Alzheimer's disease (AD) is a progressive, neurodegenerative disease that creates complex challenges and a significant burden for patients and caregivers. Although underlying pathological changes due to AD may be detected in research studies decades prior to symptom onset, many patients in the early stages of AD remain undiagnosed in clinical practice. Increasing evidence points to the importance of an early and accurate AD diagnosis to optimize outcomes for patients and their families, yet many barriers remain along the diagnostic journey. Through a series of international working group meetings, a diverse group of experts contributed their perspectives to create a blueprint for a patient-centered diagnostic journey for individuals in the early stages of AD and an evolving, transdisciplinary care team. Here, we discuss key learnings, implications, and recommendations.

## Introduction

Alzheimer's disease (AD) is a progressive neurodegenerative disease associated with high monetary costs and burden of care (1). The presentation of AD encompasses a continuum that extends from asymptomatic individuals with pathological evidence of AD (i.e., preclinical AD) (2) to patients with mild cognitive impairment (MCI) due to AD (the first clinically detectable stage of disease) and finally to patients with AD dementia (3). Pathological hallmarks of AD, β-amyloid plaques and neurofibrillary tangles, may be detectable in the brain decades before clinical symptoms appear (3). Many individuals with early-stage AD remain undiagnosed, as subtle cognitive deficits may not overtly impair activities of daily living. Subtle changes may be interpreted as normal aging by patients, family, and healthcare providers (HCPs) (4). As the disease progresses into AD dementia, symptoms of cognitive decline become more obvious, disrupt activities of daily living more frequently, and may prompt patients to seek medical attention (5).

An accurate diagnosis in the early stages of disease is critical for prognosis and advanced-care planning (1). Although an approved treatment for the early stages of AD that might delay disease progression is not yet available, a delayed AD diagnosis postpones the initiation of advanced-care planning and non-pharmacological interventions, such as cognitive stimulation, psychological treatment, and lifestyle changes that may preserve cognitive function or improve quality of life (6–9). Lifestyle modifications and enhanced social support may lessen caregiver burden, delay institutionalization, and reduce healthcare costs (10). Overall, a timely and accurate diagnosis is key to developing an effective care plan, which requires coordination between the patient, caregivers, family members, HCPs, specialists, social services, and payers (5).

The early and accurate diagnosis of AD for people in the United States born in and prior to 2018 could result in a cumulative savings of approximately $7 trillion in medical and care costs (1). Despite mounting evidence supporting the benefits of early detection (10–12) and studies indicating that most patients and caregivers would prefer disclosure of an AD diagnosis (13), the current process for diagnosis in the early stages of AD needs improvement (14). Although the recently published US Preventive Services Task Force Recommendation Statement concluded there is insufficient evidence to properly weigh the benefits and risks of screening for cognitive impairment in older adults (15), experts have been quick to contextualize these results, emphasizing the current benefits of screening for MCI and noting that approval of therapies targeting the underlying pathophysiology of AD would add further value to early screening (16).

Globally, approximately 82 million people will have dementia by 2030 at a cost of $2 trillion per year (17); 60 to 80% of these cases are likely to be caused by AD (1). To properly screen and manage the growing population of potential patients, increased resources are needed. In the current system, individuals suspected of having AD may become entangled in a cycle of continuous referrals, waiting years for a diagnosis or treatment (4). Without an early-detection paradigm in place, the already-limited infrastructure will be further strained by an influx of patients seeking treatment once a therapy targeting the underlying pathophysiology of AD is approved (14).

Interventions that target AD pathophysiology are hypothesized to be more successful when applied earlier in the course of AD, before significant neurodegeneration occurs (2, 18, 19). If such a therapy becomes available, one of the largest constraints to its use is projected to be the limited availability of specialists to evaluate and diagnose patients (14). Although the shortage of trained specialists cannot be quickly remedied, developing and implementing strategies to improve the current infrastructure and focus on patient-centric care is feasible.

To better understand how to improve the diagnostic journey of patients in the early stages of AD, a series of three international working group meetings was convened between April 2016 and May 2017. Contributors represented diverse specialties, including geriatrics, internal medicine, neurology, neuropsychology, nursing, pharmacology, and psychiatry. One-on-one interviews with contributors were conducted to gather insights from personal practice experience to identify similarities and differences in care models. These meetings were organized and held with the unrestricted support of Biogen, with an emphasis on currently available interventions and agnostic to any investigational therapy in clinical development. Here, we present the perspectives and recommendations of this group.

## Proposed Blueprint for AD Diagnosis and Care in the Early Stages of AD

**Figure 1:**
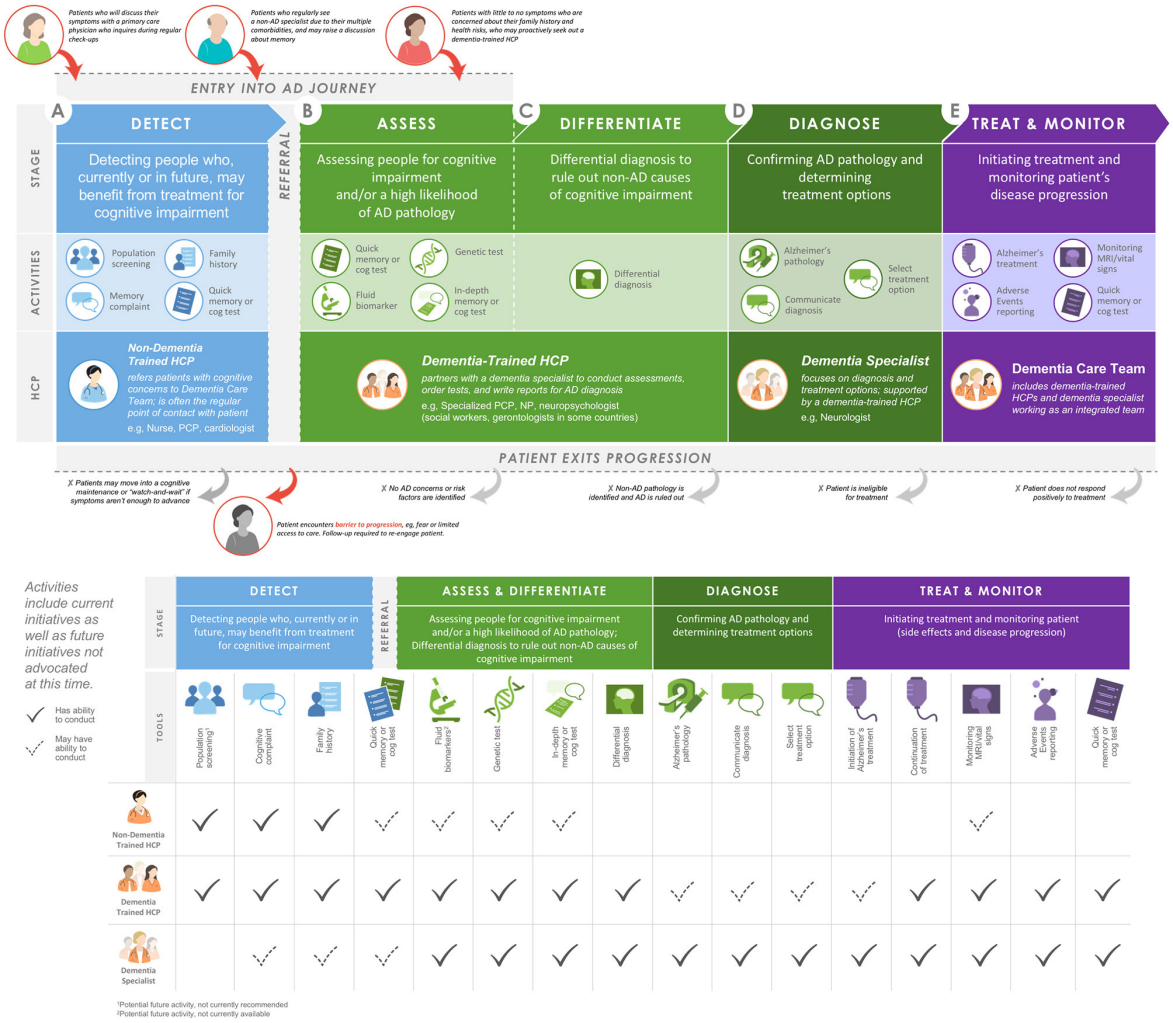
Proposed blueprint for pathways of entry into an integrated AD care team and potential roles of HCPs within the AD care team. AD, Alzheimer's disease; cog, cognitive; HCP, healthcare professional; MRI, magnetic resonance imaging; NP, nurse practitioner; PCP, primary care physician.

Through an iterative meeting process, the working group came to the consensus recommendation to establish a patient-centered diagnosis journey for individuals with early-stage AD (Figure 1). This journey is comprised of the following five stages: detect, assess, differentiate, diagnose, and treat and monitor. The diverse HCP working group applied key learnings and implications from its collective insights to create a blueprint for an evolving, transdisciplinary care team to support this diagnosis journey (Figure 1).

In clinical practice, the term “screening” is used by patients and other HCPs to broadly refer to many types of tools and practices that aid in making a diagnosis at an early disease stage. The working group realized that screening needed to be subdivided based on which HCPs and tools were likely to be involved. The initial *detection* stage may occur in a variety of scenarios where HCPs are made aware of cognitive concerns or first signs of cognitive impairment. The subsequent stages, *assessing* individuals for cognitive impairment and/or a high likelihood of AD pathology and *differentiating* AD from other causes of cognitive impairment, will require more specialized training. To fulfill this need, the working group recommends the introduction of an AD-specialist role across a variety of disciplines to diagnose early-stage AD. Following *diagnosis* of AD via biomarker testing and determination of treatment options by a dementia specialist, *treatment and monitoring* of patients may be entrusted to a patient-centered, transdisciplinary team with a variety of expertise and skills to meet the needs of the patient and caregiver.

This manuscript provides a working template for how a clinical team may operate at each step of diagnosis with active suggestions to implement training and build a collaborative team infrastructure. We identified three important focus areas (Table 1) central to achieving this, including: (a) increasing awareness of the benefits of diagnosis in the early stages of AD; (b) developing patient-centered support via the integrated AD care team blueprint; and (c) strengthening infrastructure to create the processes and capacity required to create these care teams. The implementation of this paradigm may not only improve current patient care, but also prepare our healthcare system for the anticipated increasing number of patients with AD who seek care following the approval of one or more investigational therapies targeting the underlying pathophysiology of AD (20, 21).

**Table 1:** Focus areas to improve AD care.

**A. Focus area 1: establish awareness**				
	**Raise awareness of clinical value for early detection**	**Educate about what to look for during early detection**	**Train nondementia-focused HCPs to act for their patients with early-stage AD**	**Design an ideal process to detect patients with early-stage AD**
Tactics	•Build awareness in the general population, including with patients and care partners • Educate non-dementia-trained HCPs about the clinical value of early detection	•Collect data from population databases and registries to identify risk factors and detect early-stage AD • Collect individual longitudinal data	•Train HCPs to proactively detect and support the patient • Train HCPs to refer the patient to a dementia care team	•Follow an accessible, step-by-step process, designed to stratify patient risk and help HCPs determine if a patient continues to assessment • Patient questionnaire: Assess for risk of cognitive impairment • Risk stratification: Build risk profile for cognitive impairment • Care team refer: Referrals per cognitive impairment profile
Guidance and examples	•AD experts to promote broad communication and disseminate available materials • Partner with large specialty centers and geriatric groups • Targeted information for specific subpopulations • Leverage web-based, nontraditional, and established channels	•Include information such as age, sex, race, education, and norms for each patient in databases for context • Must caution that outcomes can vary • Could support future mobile monitoring	•Train HCPs in diagnosis (including cognitive tests and available biomarkers) • Provide clear guidance for HCPs regarding current reimbursement codes • Characterize the HCP as a sentinel to refer patients to specialists • Provide referral analysis and support • Be available for outreach, tools, and communication support	•Appeal to policy makers to design new reimbursement codes focused on early detection of disease with feasible requirements for primary care providers
**B. Focus area 2: develop patient-centered support**				
	**Create a care coordination team to help patients navigate their journey**		**Increase access to services and information to increase patient engagement**	
Resources required	•Training programs or certifications within clinic or larger healthcare system, such as a specialized program for interested nurses • Initiatives to impact policy around paying for these changes; can use grants for some clinics • Large group of volunteers, either intrinsically motivated or leveraging those already in settings like nursing homes, to disseminate nonexpert info • A patient resource toolbox • A clear economic case for additional resources		•Existing or new web platform • Designated individuals to respond to questions and proactively contact concerned patients and care partners (care coordinator; see previous recommendation)	
Potential impact	•Eases patient and care partner stress associated with the diagnostic process (leading up to and after diagnosis) • Helps destigmatize AD • Empowers patients to seek clinics and care • Increases the quality of patient-to–healthcare system conversations, ensuring that the right information gets to patients when they need it • Increases capacity of HCPs • Improves patient adherence to recommendations		•Eases patient and care partner stress associated with the diagnostic process (leading up to and after diagnosis) • Helps destigmatize AD • Empowers patients to seek clinics and care; positions patients to be their own advocates • Increases the quality of patient-to–healthcare system conversations, ensuring that the right information gets to the right patient at the right time • Protects patients from misinformation/predators by giving them increased access to quality information • Increases number of potential patients with AD in the system receiving optimal care • Increases detection of early-stage AD • Increases capacity of HCPs • Decreases patient dropout rates and improves adherence and persistence	
Guidance and examples	**In a comprehensive clinic:** • Designated person within clinic to help patients navigate journey • Care coordination team may be a certified volunteer group **In an individual practice:** • Designated person(s) outside clinic (e.g., a network of RNs) who are covered via Medicare • Group-setting sessions with a local volunteer HCP to address general AD questions • Lunch and learns with willing practitioners to address concerns		•Online assessment tools and questionnaires on major AD-related websites (e.g., Alzheimer's Association) • A checklist of steps or touch points within a patient journey detailing what is required from the patient at each step to increase patient confidence • A forum on national, association-supported portals where submitted questions are answered by care coordinators • Name and contact information fields to facilitate follow-up • Peer-support or mentoring programs for new patients (e.g., similar to oncology)	
**C. Focus area 3: build processes and capacity for integrated care teams**				
	**Establish a value case to develop cross-functional teams in clinic**		**Educate future dementia-trained HCPs**	
Key considerations	•A business case to generate senior management support and buy-in, including the following: •Direct and indirect costs •Avoided costs •Holistic patient cost •Patient and care partner satisfaction •Outcomes • An understanding of different types of patients or cases and how they might experience the care model • Decision on inclusion of research component • Communication of full scope of services for the broader community • Clear information flows within the clinic and with external stakeholders • IT systems that facilitate cross-team communications • More efficient intake options, potentially sending patients packages with tests before their visit so clinic staff can appropriately identify whom patients should see or if patients are appropriate for the clinic • Triaging of patients to the appropriate team resource • A list of resources and people providing relevant services • Informational and decisional support		•Curriculum that includes computer-based tools, neuropsychology tests, and information on conducting LPs • Interactive video training modules on AD case finding and diagnosis that NPs/PAs can use • CME courses for specific diagnosis-related topics • Annual congresses with dementia-trained HCPs to standardize and share best practices • Membership to professional association to share best practices • Dementia certifications	
Potential impact	•Better outcomes for lower cost • Increased efficiency • Lower total costs when considering patient health in its entirety • Increased readiness for early-stage AD management		•Larger base of dementia-trained HCPs who can diagnose dementia	

*AD, Alzheimer's disease; CME, continuing medical education; HCP, healthcare professional; IT, information technology; LP, lumbar puncture; NP, nurse practitioner; PA, physician assistant; RN, registered nurse*.

### Detect

Detecting early stages of AD (e.g., MCI due to AD and mild AD dementia) in the clinic may occur in a variety of scenarios (Figure 1A). Some proactive patients with a family history of dementia but no evidence of symptoms or those with subjective cognitive impairment may seek a dementia specialist on their own. However, cognitive symptoms are often detected in other scenarios: in patients' discussions about memory with an HCP at annual wellness visits or with non-AD specialists treating other comorbidities. These discussions may be limited due to a patient's reticence to discuss minor cognitive complaints, the common belief that cognitive decline is a normal part of aging, and/or an HCP's limited time, training, or resources to routinely screen for cognitive impairment. Although mechanisms for reimbursement of cognitive screening exist (e.g., the Medicare Annual Wellness Visit), the prevailing pattern suggests that few HCPs take advantage of these options (5). Patients with AD are highly diverse, and the nuanced symptoms of the disease, paired with diverse family histories, comorbidities, cultural beliefs, and socioeconomic backgrounds, lead to variable entry points into the AD care system. To facilitate points of entry into early-stage AD care, the working group agreed that increasing awareness and identification of early-stage AD is imperative and composes Focus Area 1 (Table 1A).

In the general population, understanding that management options currently exist for AD may motivate more patients to discuss cognitive complaints with their HCPs or families to encourage their loved ones to discuss these problems with their providers. For non-dementia specialists, a deep understanding of the potential benefits and barriers to early diagnosis, as well as the available options for individuals with early-stage AD, is critical to facilitate early-stage AD detection. We recommend 4 priorities requiring resources and action to improve detection of early-stage AD (Table 1A). First, awareness of the clinical value of early detection in the general population must be accomplished through education about AD and its treatment options to empower patients to take control of their brain health. Second, increased education for HCPs on the clinical features of early-stage disease is imperative. This includes training clinicians to prompt discussion of cognitive signs and symptoms with their patients, make use of available dementia screening instruments that provide a valid and reproducible way to evaluate patients, recognize the predictive value of subjective concerns, and not dismiss memory complaints from the patient or family members who know the patient well. This process will be bolstered by the accumulating data on normal vs. abnormal cognitive aging that help to identify risk factors and better characterize the patient with early-stage AD. Third, non-dementia specialist HCPs should be trained to take appropriate action early. To create confidence in available resources and how to deliver care, HCPs will need additional formal training on the process of AD diagnosis. Clear guidance should be provided along with practical considerations, such as tools for conducting cognitive assessments, including the design of new reimbursement codes focused on early detection of disease with feasible requirements for primary care providers. Together, this approach may motivate HCPs to embrace their role in protecting the cognitive health of their patients by having discussions about memory and making subsequent referrals to dementia specialists. Finally, an accessible, step-by-step process to stratify patient risk and identify patients with early-stage AD should be adopted to assist HCPs in determining when further assessment or referral is appropriate.

### Assess

Currently, when cognitive impairment is identified at a primary care appointment, not all patients are referred to a dementia specialist, in part due to the national shortage of specialists such as neurologists, geriatricians, and geriatric psychiatrists (4, 22). To enable HCPs and other specialists to efficiently recognize potential cognitive impairment and administer screening assessments (Figure 1B), HCPs should have access to continuing education and diagnostic tools. To increase capacity, the working group advocates a new subspecialization of “dementia-trained HCPs.” Dementia training may be appropriate for a variety of HCPs, including internists, family medicine physicians, nurse practitioners, neuropsychologists, and physician assistants, to work in conjunction with a dementia specialist and alleviate capacity constraints. Dementia-trained HCPs could assess cognitive impairment and AD risk by administering a validated cognitive assessment, possibly accompanied by genetic or biomarker analysis. In addition to improving capacity, newly dementia-trained HCPs would be an integral part of a transdisciplinary care team. Emphasizing the role of well-established referral networks and making full use of electronic health records may reduce communication breakdowns between providers, which represent a significant barrier to a rapid, detailed assessment of initial cognitive concerns.

### Differentiate

Diagnoses across the spectrum of AD (e.g., MCI due to AD and mild dementia due to AD) are based on a comprehensive medical evaluation that incorporates clinical assessments and considers alternative causes of disease (1). Initial differential diagnosis begins with a detailed history and neurologic examination, followed by cognitive and functional assessments to examine memory, executive functioning, and behavior (Figure 1C). Cognitive decline due to AD pathology is often, although not always, characterized by impaired episodic memory that is not improved by cueing, which may normalize impaired episodic memory in other dementia diagnoses (18). Identification of a cognitive profile suggestive of impairment due to AD is followed by further assessments to rule out non-AD causes of cognitive impairment, such as bloodwork analysis for vitamin B12 or thyroid hormone deficiencies and magnetic resonance imaging for detection of tumors, stroke, head injury, or pathological profiles consistent with non-AD dementia (1). During this process, patients may become overwhelmed. The working group agreed that it was imperative to develop patient-centered support within the clinic, outlined in Focus Area 2 (Table 1B).

Establishing designated care partners, or coordinators, within the care team may ease patient stress associated with the diagnostic process. Patient-centered collaborative dementia care has been shown to improve patient stress level and mood and to reduce depression while improving caregiver confidence (23, 24). The care coordinator role may be filled by trained volunteers, social workers, or nurses. Coordinators could help manage patient expectations by providing support services to assist with access to care, improve patient education, help navigate treatment options, connect to support groups and other community resources, and understand other legal and social support available to the patient.

Disseminating information to increase engagement within the community is essential to prevent patients from abandoning the process of early-stage AD diagnosis (Table 1B). The working group posits that, along with designated care coordinators within clinics, digital screening approaches may empower patients for whom seeing a doctor may be uncomfortable or logistically infeasible. Further community education may help to destigmatize AD by eliminating the perception that an individual with AD is elderly or sick. Additionally, the design of reimbursement codes that expand upon current coverage to include a diagnostic workup aimed at differentiating AD from other cognitive disorders may expand the value case for early-stage diagnosis of AD.

### Diagnose

As understanding of AD biomarkers grows, the 2018 update of the National Institute on Aging and Alzheimer's Association research framework for AD diagnosis recommends the use of biomarkers along with clinical criteria (3) to more accurately stage AD (Figure 1D). Currently, a positron emission tomography (PET) scan is the only US Food and Drug Administration–approved diagnostic biomarker test for AD (14). Availability and increased use of cerebrospinal fluid biomarkers of AD may reduce PET imaging facility capacity constraints (14, 25). Recent granting of US Food and Drug Administration breakthrough status may increase the use of cerebrospinal fluid (26) and plasma (27) biomarkers for AD diagnosis. Additionally, interest in leveraging mobile and wearable digital consumer technology to facilitate early diagnosis of AD is growing (28).

### Treat and Monitor

Currently, where a patient with AD enters the healthcare system greatly influences the care they receive (Figure 1E). Variation in available management and treatment approaches, including access to dementia specialists, neuroimaging facilities, and support groups, is complicated by both geography and financial resources. In rural and resource-limited areas, added emphasis on training, both live and virtual, of non-dementia specialists is vital. The working group recommends building and strengthening the integrated dementia care team infrastructure as Focus Area 3 (Table 1C).

The working group acknowledges that for networks and healthcare systems to be motivated to implement integrated AD care teams, a strong and clear value case is required to justify adapting the proposed framework and allocating additional resources (Table 1C). The large-scale, interventional Finnish Geriatric Intervention Study to Prevent Cognitive Impairment and Disability (FINGER) trial and *post-hoc* analyses of the Multidomain Alzheimer Preventive Trial (MAPT) and Prevention of Dementia by Intensive Vascular Care (PreDIVA) trial indicate that multidomain lifestyle interventions for AD prevention may have a beneficial effect in slowing cognitive decline in at-risk populations, bolstering the value case for early intervention even in the absence of a therapy for the early stages of AD (10, 29–32). In addition to buy-in from key financial stakeholders and the AD healthcare community, practical infrastructure must be established to create AD care teams. To address capacity barriers, a program that paired dementia specialists with dementia-trained HCPs would be the basis of a broader AD care team (Table 1C) that supports implementation of strategies to prevent further cognitive decline and provide immediate access to symptomatic therapies and future disease-modifying therapies.

## Looking Forward

The numerous unique pathways to AD diagnosis and treatment motivated the working group's recommendation for a tailorable, patient-centered care environment. Indeed, the model presented here is intended to be optimally efficient but also significantly flexible to accommodate a real-world setting. We advocate for HCPs to work as an integrated care team to accommodate the diverse population of patients with AD, who have varied entry points to screening and treatment. The successful implementation of a patient-centered, single-entry-point, no-wrong-door model would help identify patients at risk for cognitive decline and guide patients into care through multiple initial points of contact (33, 34). A no-wrong-door model would further streamline AD pathology assessment and differential diagnosis through the cooperative efforts of public agencies, outreach organizations, HCPs, payers, and specialists to ensure that early warning signs of AD are not overlooked. This patient-centric, transdisciplinary care model to diagnose and treat patients early may also have applicability in other diseases with large unmet needs, such as cerebrovascular disease and Parkinson's disease.

A limitation of the working group recommendations is the focus on US care models. These recommendations will need adaptations to fit healthcare systems of other countries. However, the proposed AD care team aligns with multiple action areas of the World Health Organization's 2017 *Global Action Plan on the Public Health Response to Dementia*, which emphasizes the need for dementia awareness, risk reduction, and support for those with dementia and their caregivers throughout treatment (35). Adopting this blueprint would also provide a better framework and increased capacity to apply the 2015 Gerontological Society of America working group–recommended Kickstart, Assess, Evaluate, Refer (KAER) model to increase detection of cognitive impairment and improve outcomes for Medicare beneficiaries (4).

We recognize the immense challenge of implementing large-scale, systemic changes, which will require the cooperation of HCPs, specialists, patients, caregivers, outreach organizations, and financial stakeholders. Although securing additional resources to raise awareness of early-stage AD and developing infrastructure for a patient-centered AD care team are paramount to supporting patients and their care partners, many barriers remain. AD must be destigmatized so patients can seek and understand the benefits of early detection and intervention while gaining access to early diagnosis without discrimination. Addressing such barriers will allow patients to optimally navigate AD diagnosis and disease management.

## Data Availability Statement

The original contributions presented in the study are included in the article/supplementary material; further inquiries can be directed to the corresponding author/s.

## Author Contributions

All authors attended and contributed to the international working group discussions. All authors contributed to the final manuscript.

## Conflict of Interest

JG has been a paid consultant for Biogen. PA reports grants from, NIA, FNIH, the Alzheimer's Association, Janssen, Lilly, and Eisai, and personal fees from Merck, Roche, Biogen, Lundbeck, ImmunoBrain Checkpoint, and Samus. JL reports grants from the National Institutes of Health (NIH/NIA), Alzheimer's Association, FBRI, Novartis, and Roche. ER received assistance from Biogen with conference expenses including honorarium and travel/lodging expenses in May 2017. MS has intellectual property rights (royalties or patent sales with Harper Collins. He has ownership interest (stock, stock options) with Versanum Inc, Brain Health Inc, Optimal Cognitive Health Company, uMethod Health, and NeuroReserve. He has been a consultant for Biogen, Signant Bracket, Eisai, Alzheon, Athira, NeuroReserve, Stage 2 innovation, vTv therapeutics, Regeneron, Neurotrope, and Roche-Genentech. RS is a speaker for Biogen and a Medical Director for EVERSANA, which does work for Biogen. RAS received consulting fees as a member of the ADvance International Working Group, which was unrelated to the current manuscript. He has stock options as a member of the Board of Directors of King Devick Technologies. He has received royalties from Psychological Assessment Resources, Inc for published neuropsychological tests. He has received consulting fees from NCAA Concussion Settlement as a member of the Medical Scientific Committee. EV has received grant support from Eli Lilly, Biogen, Hoffman-LaRoche, Cognito Therapeutics, Eisai, National Institutes of Health (NIH/NIA), Cortexyme, and Avanir. She is a consultant for Biogen and Cognito Therapeutics. NW was an employee of Biogen at the time of this work. IR is an employee of Biogen. The remaining author declares that the research was conducted in the absence of any commercial or financial relationships that could be constructed as a potential conflict of interest.
